# Intelligent Controller Design by the Artificial Intelligence Methods

**DOI:** 10.3390/s20164454

**Published:** 2020-08-10

**Authors:** Jana Nowaková, Miroslav Pokorný

**Affiliations:** 1 Department of Computer Science, Faculty of Electrical Engineering and Computer Science, VSB-Technical University of Ostrava, 17. listopadu 2172/15, 708 33 Ostrava – Poruba, Czech Republic; 2 Department of Cybernetics and Biomedical Engineering, Faculty of Electrical Engineering and Computer Science, VSB-Technical University of Ostrava, 17. listopadu 2172/15, 708 33 Ostrava – Poruba, Czech Republic; miroslav.pokorny@vsb.cz

**Keywords:** intelligent controller, PID controller, artificial intelligence, expert systems, fuzzy methods, genetic algorithms, optimization, softcomputing

## Abstract

With the rapid growth of sensor networks and the enormous, fast-growing volumes of data collected from these sensors, there is a question relating to the way it will be used, and not only collected and analyzed. The data from these sensors are traditionally used for controlling and influencing the states and processes. Standard controllers are available and successfully implemented. However, with the data-driven era we are facing nowadays, there is an opportunity to use controllers, which can include much information, elusive for common controllers. Our goal is to propose a design of an intelligent controller–a conventional controller, but with a non-conventional method of designing its parameters using approaches of artificial intelligence combining fuzzy and genetics methods. Intelligent adaptation of parameters of the control system is performed using data from the sensors measured in the controlled process. All parts designed are based on non-conventional methods and are verified by simulations. The identification of the system’s parameters is based on parameter optimization by means of its difference equation using genetic algorithms. The continuous monitoring of the quality control process and the design of the controller parameters are conducted using a fuzzy expert system of the Mamdani type, or the Takagi–Sugeno type. The concept of the intelligent control system is open and easily expandable.

## 1. Introduction

Sensor-based measuring systems are used not only in the information systems but also in the control ones. Quality control of real complex processes requires continuous monitoring of the changing internal and external conditions, followed by continuous adaptation of the controller parameters. Nowadays, sensor networks are widely used and easily available. We are facing a situation that the sensors provide us with even more information that we are able to use. We can work with a really massive amount of data–big data is trying to find hidden context in data, and the next step is to use the information contained in the data. The world of processes controlling should also change. It must reflect the situation of the enormous amount of data obtained from the processes as well as being able to work with it. The main idea of the work is to present a non-conventional method of solving classic control problems–how to set the controller parameters and when and how to reset them.

The controlling system design for continuous adaptation under time changing conditions is often discussed in many application issues.

Up to the present time, many methods and controlling schemes have been developed. There is a large group of classic design methods [1], techniques based on artificial intelligence [2], or ideas built on a combination of traditional and non-conventional methods. The combination of the methods exploits the best both of these methods provide. The fact that the use of non-conventional methods offers the possibility to add inputs and rules, which are elusive for the standard methods, is one of the biggest advantages.

The proposed solution of the controlling system combines continuous adaptation of the PID controller, monitoring of control process quality according to the predetermined requirements in real conditions of dynamically changing attributes of a controlled system, and ongoing identification of the controlled system from the sensor data. The sensitive and accurate sensors and memory for the data measured form the necessary parts of the system.

### 1.1. Related Work

Process controlling is a broad scientific field involving various areas. The essential areas of interest comprise intelligent controlling including its parts–control quality monitoring, system identification, and controller parameter adjustment, which are designed using artificial intelligence techniques. The perspective may be formulated as the use of expert systems in controlling and the use of non-conventional optimization methods in controlling. These two groups were developed simultaneously, and several mixed (hybrid) algorithms are also proposed.

As stated in [2], there are two approaches in controlling using an expert system (ES). The first one is the fuzzy rule-based method for controlling processes for which suitable models do not exist or are inadequate. The rules substitute for conventional control algorithms. The second method, originally suggested in [3,4], involves the use of an ES to extend the amounts of classic control algorithms.

It was 1984 when Moore et al. [5] introduced the use of an ES in industrial applications. The system was designed to monitor a process loop of a conventional control system with an intelligent alarm system.

In 1986 Äström, Anton and, at the time Äström’s Ph.D. student, Ärzén [4], presented an idea to replace heuristic logic with an expert system in the feedback loop in a classic controlling issue. This has led to a simplification of heuristic logic and has brought new functionalities usable in control systems. A classic PID is considered as the controller. They propose some ideas on how to design a monitoring block and PID controller parameters. This proposed approach can be regarded as the first mention and a basis for expert control, where all the parts are knowledge-based.

Shirley [6] describes the experience with the use of the ES in controlling, namely in controlling an adipic acid reactor and a heat exchanger. The limits of the ES, which should not be considered, according to the author, as a panacea, have been mentioned. From the author’s point of view, expert systems are not experts; it means that the ES does not have all the data that an expert could have. Currently, great attention is paid to the ES. The ES should be chosen according to the specific application. The costs of the ES are declining and acquiring knowledge from experts should be conducted with care, with the consciousness that computer power and memory are finite. General rules could be more powerful than more specific rules, and there is a necessity of “good” data. The essence of the article is that Shirley was sceptical about using the ES.

Nowadays, 30 years later, the situation has changed. Based on our work, it can be asserted that the issue of intelligent controllers has been experiencing a renaissance in the last three or four years. One group of PID controller parameters optimization and setting approach uses ant colonies, genetic algorithms, and artificial neural networks [7]. The procedure, which the authors also call intelligent controlling, involves using a single adaptive neuron–direct neural control [8] with a hybrid learning based on the conjunction of an on-line rule based on the gradient descent method and an off-line rule based on the Big Bang-Big Crunch algorithm. A two-layer feed-forward neural network with as few neurons as possible with two inputs, *e* and Δe, is also proposed. The output is in the form of a change in the duty cycle for the motor control [9].

Non-conventional approaches have become established in industrial applications in the last few years. Controlling of a hydraulically driven robot based on the implementation of two schemes is presented in [10]. The first scheme is created using a fuzzy-PID self-tuning controller composed of the conventional PID control and including fuzzy logic. The second one consists of the adaptive neuro-fuzzy inference system-PID (ANFIS-PID), self-tuning of the gains of the PID controller. However, the fuzzy logic controller [11] or the smart method of controlling [12] (and many others) are very often confused for the intelligent controller.

Conventional PID controller parameters can also be set [13] by the ES of the Mamdani type with two inputs–*e* and Δe and three outputs–$\Delta k_{p}$, $\Delta k_{i}$ and $\Delta k_{d}$. The knowledge base consists of 49 easy rules for each output. An almost identical approach was selected for the PID controller in the photovoltaic system [14]. The other one, the Sugeno type system, extends it, and it is used for optimization and shape definition of input variables membership function. Next, another Sugeno type of the ES for PID controller parameters determination is also proposed.

An effortless and user-friendly method of using decision-based controlling for Smart Home is proposed in [15]. A combination of an artificial neural network and the fuzzy logic controlling system can be devised for a switched reluctance generator, but the controlling systems work independently, without any connection [16].

An exciting implementation of Brain Emotional Learning Based Intelligent Controller is described in [17]. It is proposed for the coordination of multi-agent systems and based on emotional learning in the mammalian limbic system.

Bigdeli and Ziazi [18] designed a robust fractional-order adaptive intelligent controller devised for the stabilization of uncertain fractional-order chaotic systems. The authors prepare the intelligent neuro-fuzzy network for unknown dynamics estimation of the system, while the neuro-fuzzy network parameters, upper bounds of the model uncertainties, disturbances, and approximation errors are adaptively estimated via separate adaptive rules.

The authors of [19] set the goal to improve the control performance for the uncertain nonlinear systems. The idea of the study is to use a self-organizing, fuzzy neural network to imitate the control law directly and, then, to appeal to obtain a compact structure of the controller to further reduce the computational burden and to enhance the control performance.

Controlling system tuning by an online fuzzy-based approach was proposed to intelligently optimize the gain values in the load frequency control structure in [20]. Then the intelligent fuzzy based inference system for autonomous mobile robot navigation is presented by [21], for a rigid robotic manipulator system, it is presented by [22] or, for bicycle robot balancing, it is presented by [23].

System identification is also a necessary task for the design of the appropriate controlling system. This is an estimation of the system parameters from the data observed. There are a lot of classic system parameters identification methods, such as nonparametric time- and frequency-domain or estimation methods [24,25]. In the identification area, some problems can be formulated as optimization tasks. They can be solved by various high-level optimization strategies employed to minimize (or maximize) an objective function representing the problem. Optimization algorithms can be, in general, divided into two main groups. The first group contains deterministic algorithms, while the second one comprises stochastic (randomized) methods. In our case, the stochastic method–genetic algorithms [26]–is used. Unfortunately, no mention in connection with the use of genetic algorithms in process identification can be found. On the contrary, references to the use of fuzzy clustering [27] or neural networks [28,29] can be found.

### 1.2. The Features of the Proposed Solution

The proposed solution is based on soft-computing techniques, is open, flexible, and provides the possibility of expert knowledge embedding. The final intelligent controller is modular. It comprises a module of ongoing process quality monitoring, a module for processing transfer function identification and a module for setting the PID controller parameters. All the modules could be used separately as well as in other concepts for process control. The modules are designed generally for controlling a system of a higher order.

The algorithms of the artificial intelligence procedure are formally as simple as possible, understandable for the user and usable in the area of education of new engineers for their clarity. This type of solution minimizes the requirements for computing performance and is suitable for the use in embedded systems. The simulation experiments are selected to prove the efficiency of the non-conventional methods in comparison with the conventional ones.

The main idea is to effectively combine the non-conventional methods with the classic ones and to present the possible use. Such a solution allows the use of a combination of exact measurements and vague expert knowledge to improve monitoring and control procedures. It has been decided to combine two approaches to the artificial intelligence area. The first one is the use of the fuzzy-logic rule-based models (expert systems) for the quality improvement of continuous monitoring of the controlling process from the sensor data. This fuzzy system is of the Mamdani type with rule-based operating under two inputs, but it can be very easily extended [30]. The output of the system is the decision if the actual quality of the controlled process is appropriate, or there is necessity to re-adapt the controlling system. The second fuzzy expert system of the Takagi–Sugeno type is proposed for the controlling system parameters design according to the decision of the controlling process quality [31]. This tool is also open as for all its aspects, and its knowledge base is built on traditional time-tested PID controller design methods. For the identification of the controlled system parameters, the genetics algorithms optimization methods are proposed. Genetics algorithms are used for finding the parameters of the difference equation of the controlled system.

When setting the solution to the state of the art of the intelligent controlling area, the proposed design is sophisticated. All the modules are designed on the basis of unconventional methods. The family of the controlling process is more extensive; it is not designed for one particular controlled system. The paper has not presented a solution for one controlling issue. Yet, it is more general, but it includes the possibility to adapt the modules to a specific issue in a simple manner.

The remaining part of this paper is organized as follows. Section 1 summarizes the work relating to intelligent controlling systems. Section 2 outlines the proposed design and details the principles of all parts of the systems presented. Section 3 provides the numerical verification and the verification by simulation to illustrate the ability of the proposed solution to find an accurate response to the changing conditions of the controlled system. First of all, the modules are presented and simulated separately to show the efficiency of every module in various cases. Then, the entire intelligent controller is simulated with the description of the re-adaptation strategy. Section 4 provides a short discussion on the related topics.

## 2. Materials and Methods

**Figure 1:**
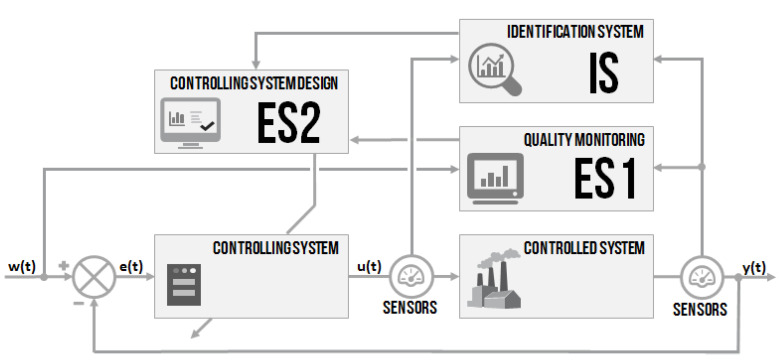
The schema of the proposed intelligent controlling system.

It has been mentioned in the beginning that the idea is to design an intelligent controlling system, where all the essential parts are built on the use of non-conventional methods. A combination of two approaches from the field of artificial intelligence–fuzzy-logic expert systems for the decision of the appropriate or non-appropriate controlling quality and for controller parameters setting–was proposed. Then, a non-conventional identification of the controlled system parameters by genetic algorithms was designed. All the parts can be used separately or together. The intention was to design systems based on fuzzy-logic principles [32] and soft-computing methods [33,34], as well as novel systems based on standard methods [35]. A simplified system block schema (Figure 1) used three modular blocks–ES1, IS, and ES2. The schema was built on the primary negative feedback loop controlling with two sensors placed for measuring the input u(t) and output y(t) of the controlled system. All the parts are going to be described in detail. The design is proposed more generally, but provides a particular example.

### 2.1. Quality Monitoring–ES1

This section focuses on the adjustment of the quality monitoring system (ES1) [30] for deciding when to re-adapt the classic PID controller. In the schema of the intelligent controller, it was connected to a block for controlling the system design. The monitoring system presented has some common elements with [36]. A fuzzy expert system [37] of the Mamdani type [32,38] was created. This system contains two inputs (relative overshoot and settling time), but the settling time is not defined as a classic time quantity, but as the part or multiple of the previously measured settling time (relative settling time), and one output (score). The score determines if it is necessary to re-adapt the controller.

**Figure 2:**
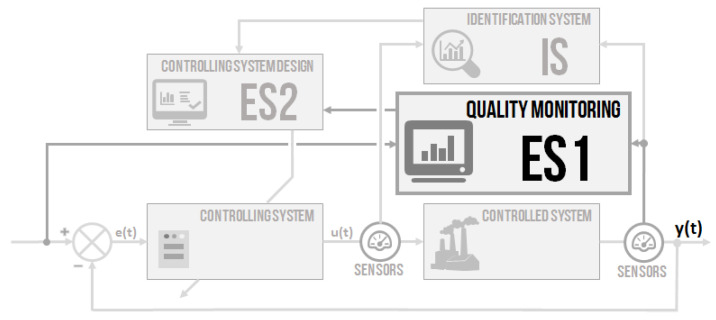
The schema of the concept of intelligent controlling system with outlined block of monitoring procedure.

To better understand the article and the issue itself, a block containing monitoring procedure on the schema of the intelligent controller is outlined in Figure 2.

#### 2.1.1. Quality Monitoring Inputs

**Figure 3:**
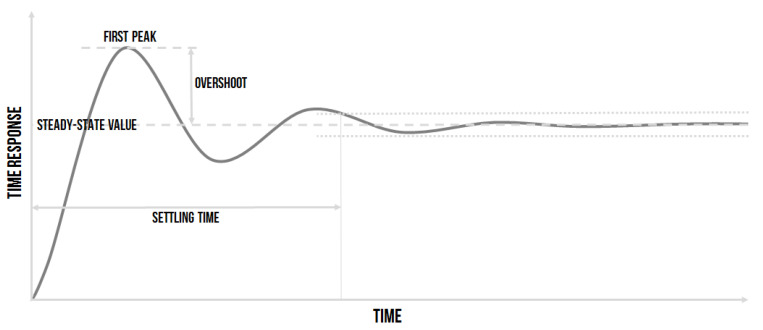
The depiction of the overshoot and the settling time in general timing.

The fuzzy expert system ES1 [30] is a decision support system [39] with two inputs, which are easily obtained from timing a controlled process–relative overshoot and relative settling time. The meaning of these variables is depicted in (Figure 3). As the relative overshoot, the first peak overshoot rated to the steady-state value of the step response was chosen. The settling time was the time from which the timing stays in the range of ±p% of the steady-state value of the system step response [1].

ES1 was the fuzzy expert system, so the inputs were the linguistic variables with the following meaning. The first input was called Relative Settling Time (RST), it was the linguistic variable with settable linguistic values. The Relative Settling Time (Equation (1)) was computed as the ratio of the actual settling time ($ST_{k}$) and the settling time of previous steady state ($ST_{k-1}$):

(1) $$RST_{k}=\frac{ST_{k}}{ST_{k-1}}.$$

It is necessary to save the settling time of the previous steady-state in the memory. The settling time is often set as a 2% standard deviation from the steady-state value [1], but the user can choose its value in our opinion, it is an optional value.

**Figure 4:**
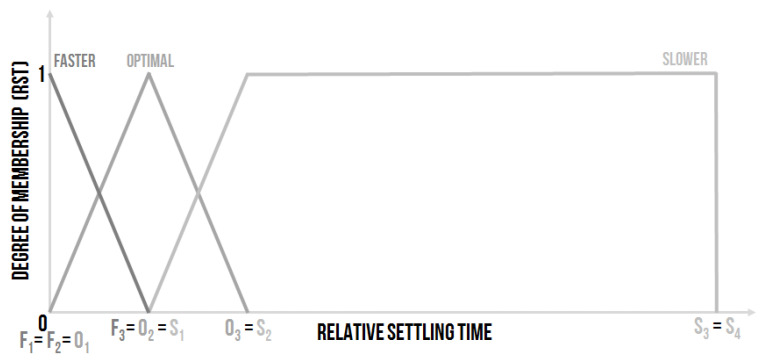
The shape of the membership functions of the linguistic values for the input linguistic variable Relative Settling Time (RST) [30].

It is proposed to set the linguistic values of the RST as follows (Figure 4):

- Faster–the settling time, in comparison with the previous settling time, is shorter
- Optimal–the settling time and the previous settling time are almost the same
- Slower–the settling time, in comparison with the previous settling time, is longer

The numerical description of the broken-line shape of the linguistic values membership functions could be provided as follows:

- Faster–$\left[F_{1}\;F_{2}\;F_{3}\right]$, where e.g., $F_{1}=0$, $F_{2}=0$ and $F_{3}=1$, it means Faster—$\left[0\;0\;1\right]$, the right border $F_{3}=1$ is equal to the idea that, in this point, the RST of previous and actual time course are the same and do not belong to this linguistic Faster value.
- Optimal–$\left[O_{1}\;O_{2}\;O_{3}\right]$, where e.g., $O_{1}=0$, $O_{2}=1$ and $O_{3}=2$, it means Optimal—$\left[0\;1\;2\right]$. The Optimal value means that the actual settling time is around the value of the previous settling time and is not greater than two times. The right border $O_{3}=2$ could be set as another value, in this case the monitoring system is not going to be so “strict”.
- Slower–$\left[S_{1}\;S_{2}\;S_{3}\;S_{4}\right]$, where e.g., $S_{1}=1$, $S_{2}=2$, $S_{3}=100$ and $S_{4}=100$, it means Slower–$\left[1\;2\;100\;100\right]$. The value of $S_{2}$ can also be set to the other value according to the value of $O_{3}$; the bigger $S_{2}$ and $O_{3}$ (proposing $S_{2}$ = $O_{3}$) are the less strict the monitoring system is. The values exceeding the limit of $S_{4}$ are considered as equal to $S_{4}$, as the limit of the linguistic Slower value.

The linguistic input called Relative Overshoot (RO) was selected as the second input of the fuzzy expert system for the quality of the time response. The value of the overshoot can be seen in the time response of the controlled system, and it is detected after any change in the closed controlling loop. The relative overshoot is represented as the difference between the maximal overshoot (MO) of the controlled variable (*y*(*t*)) and the change of the setpoint–the required value (*w*(*t*)) (see Figure 1) is rated relative to the required value (Equation (2)):

(2) $$RO=\frac{\left|MO-w(t)\right|}{\left|w(t)\right|}.$$

The relative overshoot is expressed in percentage. The value of the Relative Overshoot should be stored in the memory along with the value of the Relative Settling Time.

**Figure 5:**
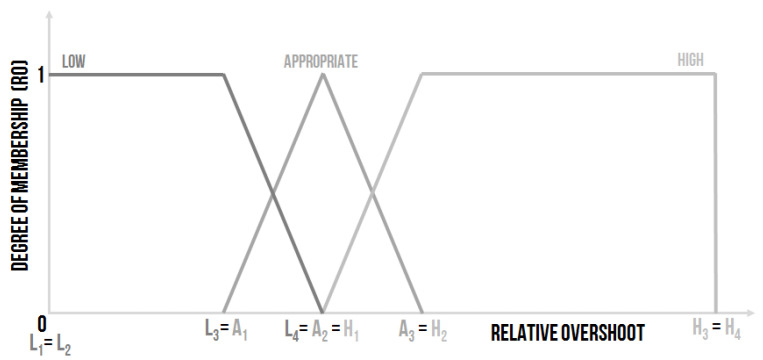
The shape of the membership functions of the linguistic values for the input linguistic variable Relative Overshoot (RO)–design no. 1 [30].

This linguistic input was settable, and the use of three linguistic values was proposed (Figure 5):

- Low–the relative overshoot is small, and, from the controlling point of view, it is a good state.
- Appropriate–the value of the relative overshoot is small enough, the border for the acceptable overshoot is often set as 20%.
- High–the relative overshoot is high, which is not good for many controlled systems and especially for the sensors (which are used for the measurement of the required value). The influence of a high overshoot on the sensors could be fatal. The sensors can be destroyed by a high overshoot, which is a better option. Or, the high overshoot can damage the sensor, but the sensor will function, although inaccurately. The poor functionality of the sensor will not be clear at first sight, and the entire system will work unpredictably.

The shape of the membership functions of the linguistic values expressed by the fuzzy sets can be written using the numerical entry:

- Low–$\left[L_{1}\;L_{2}\;L_{3}\;L_{4}\right]$, where e.g., $L_{1}=0$, $L_{2}=0$, $L_{3}=0.1$ and $L_{4}=0.15$, it means Low–$\left[0\;0\;0.1\;0.15\right]$.
- Appropriate–$\left[A_{1}\;A_{2}\;A_{3}\right]$, where e.g., $A_{1}=0.1$, $A_{2}=0.15$ and $A_{3}=0.2$, it means Appropriate–$\left[0.1\;0.15\;0.2\right]$.
- High–$\left[H_{1}\;H_{2}\;H_{3}\;H_{4}\right]$, where e.g., $H_{1}=0.15$, $H_{2}=0.2$, $H_{3}=10$ and $H_{4}=10$, it means High–$\left[0.15\;0.2\;10\;10\right]$.

**Figure 6:**
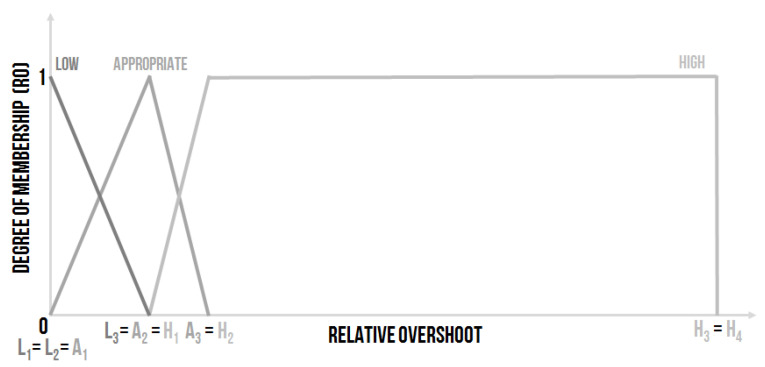
The shape of the membership functions of the linguistic values for the input linguistic variable Relative Overshoot (RO)–design no. 2.

The other possible setting could be defined as follows (Figure 6):

- Low–$\left[L_{1}\;L_{2}\;L_{3}\right]$, where e.g., $L_{1}=0$, $L_{2}=0$ and $L_{3}=0.15$, it means Low–$\left[0\;0\;0.15\right]$.
- Appropriate–$\left[A_{1}\;A_{2}\;A_{3}\right]$, where e.g., $A_{1}=0$, $A_{2}=0.15$ and $A_{3}=0.2$, it means Appropriate–$\left[0\;0.15\;0.2\right]$.
- High–$\left[H_{1}\;H_{2}\;H_{3}\;H_{4}\right]$, where e.g., $H_{1}=0.15$, $H_{2}=0.2$, $H_{3}=10$ and $H_{4}=10$, it means High–$\left[0.15\;0.2\;10\;10\right]$.

The second design proposal is not so “strict”. The design depends on the expert’s experience, the nature of the controlled system, and the method of the controlling strategy.

#### 2.1.2. Quality Monitoring Output

Expert system ES1 represents a system with two linguistic inputs and one linguistic output–Score. The Score linguistic variable must be defuzzified using the Center of Area method (COA) [33]. The higher the value of the Score is the more the controlling process is considered as good enough and the selected controller as appropriate. It was set that score value of less than “two” is regarded as unsatisfactory, and the controller has to be re-adapted [36]. Everything equalling or exceeding “two” is regarded as good enough.

**Figure 7:**
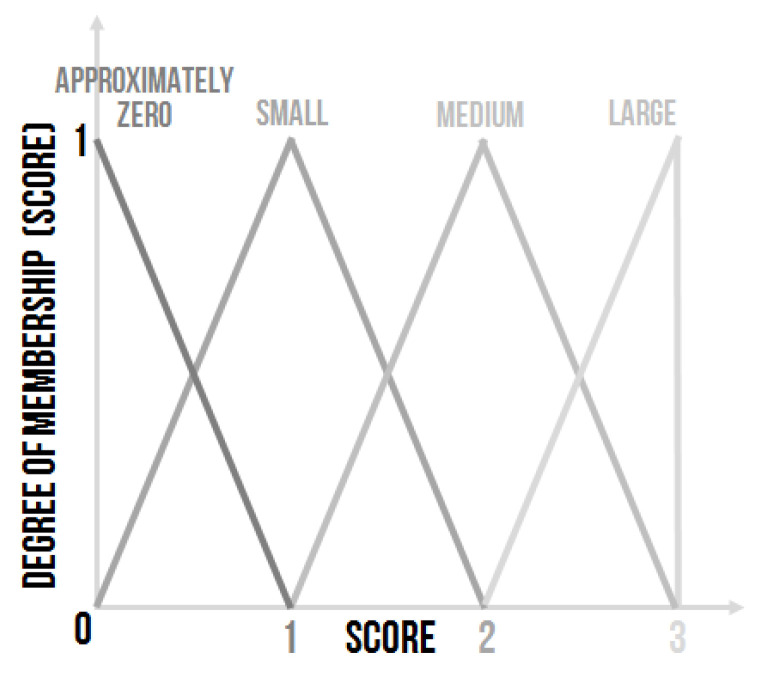
The shape of the membership functions of linguistic values for the output linguistic variable Score [30].

The Score linguistic variable is also a linguistic variable with linguistic values expressed as fuzzy sets (Figure 7). In this case, four linguistic values were used, and their triangular-shape membership functions could be described as follows:

- Approximately zero—$\left[0\;0\;1\right]$,
- Small—$\left[0\;1\;2\right]$,
- Medium—$\left[1\;2\;3\right]$,
- Large—$\left[2\;3\;3\right]$.

The linguistic values of the Score can be adjusted for a particular case if necessary.

#### 2.1.3. Quality Monitoring System Knowledge Base

The knowledge base was formed by nine linguistic IF-THEN rules of the Mamdani type [33]:

1. If (ROisLow)and (RSTisSlower)then (ScoreisMedium)
2. If (ROisLow)and (RSTisOptimal)then (ScoreisLarge)
3. If (ROisLow)and (RSTisFaster)then (ScoreisLarge)
4. If (ROisAppropriate)    and (RSTisSlower)then (ScoreisSmall)
5. If (ROisAppropriate)    and (RSTisOptimal)then (ScoreisMedium)
6. If (ROisAppropriate)    and (RSTisFaster)then (ScoreisLarge)
7. If (ROisHigh)and (RSTisSlower)then (ScoreisApprox.zero)
8. If (ROisHigh)and (RSTisOptimal)then (ScoreisSmall)
9. If (ROisHigh)and (RSTisFaster)then (ScoreisMedium)

The shape of the membership function of the output variable was inferred using the Mamdani method [33]. As the inference method, the Fuzzy Modus Ponens derivation rule was implemented [40]. The linkage between the rules in the knowledge base is disjunctive. Fuzzy conjunction and fuzzy implication were interpreted as basic logic functions—fuzzy disjunction, fuzzy conjunction and fuzzy implication were interpreted as maximum, minimum and minimum resp. According to this fact, the algorithm of the derivative methods was straightforward. The crisp value of the Score output was performed using the Center of Area defuzzification method.

### 2.2. Identification System–IS

Controlled system parameters identification is a known and necessary task in controlling the design and its following adaptation [41]. System identification is used to assess the nature of the systems. The use of genetic algorithms, as the optimization procedure for finding the parameters of the system transfer function, is proposed. The system representation in the form of the transfer function was selected because it was available from the sensor data. It was suitable for a black-box description, and it was easy to use for our next step—controlling the system adaptation, if necessary. An identification system (IS) is generally designed to be connected to ES2 in the intelligent controller schema. The information on the ongoing identification is necessary for the re-adaptation of the PID controller parameters. The method presented can be used both for continuous and discrete systems [42,43].

The idea of the system prepared was to identify the parameters of the difference equation from the sensors data–the input–output values of the system measured (system input-control variable, system output). It was built on the optimization feature of genetic algorithms (GA) [44]. In our case, GAs were used to find the coefficients of the difference equation of the controlled system according to the selected fitness function.

**Figure 8:**
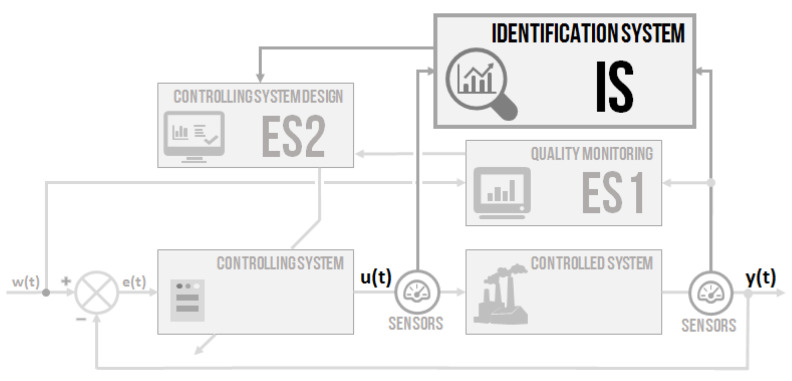
The schema of the concept of intelligent controlling system with outlined block of the controlled system identification procedure.

The block containing the identification procedure on the schema of the intelligent controlling system is outlined in Figure 8.

The measurements (sensor data evaluation) were always performed in some time-points (predefined)–with a preset period (sampling period) *T*. The fact that the sampling period should be small enough to be able to reconstruct the signal according to the Nyquist–Shannon sampling theorem [45] should be taken into consideration.

The identification aims to fit the mathematical model as well as possible and to eliminate the influence of the noise distortions to the greatest extent [41]. As for the proposed method of identification, the presence of additive noise could not affect the result of the identification severely. In reality, the more variable the input signal is the better results could be obtained. Nevertheless, the noise level should not be too high; it means that the signal-to-noise ratio should be big enough.

The identification procedure could be used for any system for which the transfer function can be written. At the beginning, the expert should define the system order according to the nature of the controlled system. Hence, the expert should determine, for example, that the system is of the third order or the system can be identified as a system of, for example, up to the fourth order with the decision according to the accuracy needed. The effectiveness of the identification procedure for the system of the third order is going to be described in Section 3, but we suggest that the second order is sufficient because ES2 is designed for the system of the second order. The only thing which should be borne in mind is that the higher the order of the system is found the longer the computing time/greater computing power is required. Therefore, in general, the system order is not limiting; the only limit is the required time and the computational equipment. As for the second-order system, four unknown values must be found; as for the third-order system, six values must be found; and, as for the fourth-order system, eight values must be found, etc.

Genetic algorithms (GAs) have been one of the first and most popular evolutionary algorithms. GAs are based on computer implementation of biological population-based evolution with selection, mutation, and crossover principles [44].

The third-order system was selected as an example of the use and it can be defined using the transfer function in the following form:

(3) $$G_{S}\left(s\right)=\frac{b_{3}s^{3}+b_{2}s^{2}+b_{1}s+b_{0}}{a_{3}s^{3}+a_{2}s^{2}+a_{1}s+a_{0}}.$$

The difference equation is then defined as:

(4) $$\begin{array}{ccc} y\left(k\right)= & - & \frac{a_{k-1}}{a_{k}}y\left(k-1\right)-\frac{a_{k-2}}{a_{k}}y\left(k-2\right)-\frac{a_{k-3}}{a_{k}}y\left(k-3\right)\\ & + & \frac{b_{k-1}}{a_{k}}u\left(k-1\right)+\frac{b_{k-2}}{a_{k}}u\left(k-2\right)+\frac{b_{k-3}}{a_{k}}u\left(k-3\right), \end{array}$$

with

(5) $$\begin{array}{ccc} Y_{1} & = & \frac{a_{k-1}}{a_{k}},\\ Y_{2} & = & \frac{a_{k-2}}{a_{k}},\\ Y_{3} & = & \frac{a_{k-3}}{a_{k}},\\ U_{1} & = & \frac{b_{k-1}}{a_{k}},\\ U_{2} & = & \frac{b_{k-2}}{a_{k}},\\ U_{3} & = & \frac{b_{k-3}}{a_{k}}. \end{array}$$

where $Y_{1},Y_{2},Y_{3}$ and $U_{1},U_{2},U_{3}$ are the optimized parameters for which the GA are used.

For finding of the coefficient of difference equation the fitness function has to be defined, for our solution the fitness function is defined as:

(6) $$J=\frac{1}{N}\sum_{j=1}^{N}{\left[y^{measured}\left(x_{j}\right)-y^{GA}\left(x_{j}\right)\right]}^{2}\to 0,$$

where *N* is the size of the set of input-output data.

### 2.3. Controlling System Design–ES2

The entire proposed solution would not be complete without the last part—the controller parameters design system (ES2). Three versions of this module were designed, wherein the know-how obtained from the classic PID controller design methods was implemented. The knowledge from the Ziegler–Nichols (ZN) step response method, a combination of Ziegler–Nichols methods (the combination of the frequency response method and the step response Ziegler–Nichols design method) [46] and one of the possible modification of the Ziegler–Nichols step response method–the Chien, Hrones, and Reswick (CHR) setpoint response design method with a 0% overshoot [1] in a variant with a 0% overshoot (for more information see [31]), was applied.

**Figure 9:**
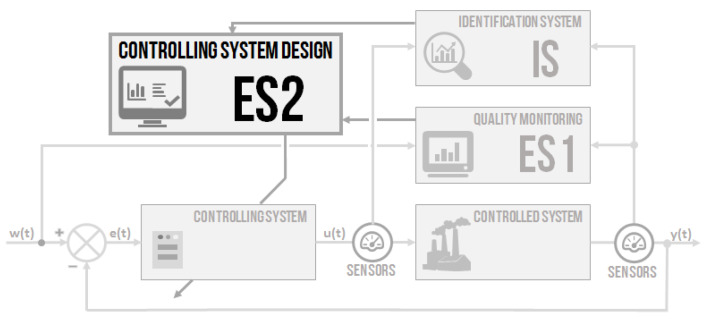
The schema of the concept of intelligent controlling system with outlined block of the controller parameters design system.

ES2 is a fuzzy expert system based on a model of the Takagi–Sugeno type without the necessity of the defuzzification procedure [37]. The block containing ES2 on the schema of the intelligent controlling system is outlined in Figure 9. ES2 was connected with the IS and ES1. The connection with the IS provided information about the structure and parameters of the controlled system. The relationship with ES1 provided the information if the re-adaptation of the controller parameters was required or not.

#### 2.3.1. Controlling System Design Inputs

The proposal was to diversify the working area of the controlled system to smaller parts and to design, for every part, the appropriate controller using the classic design controller method and the parameters obtained from the PID controller used for the knowledge design. The method should be selected by the expert. A demonstration was conducted for the system of the second order with the following transfer function:

(7) $$G_{S}\left(s\right)=\frac{1}{a_{2}s^{2}+a_{1}s+a_{0}}.$$

**Figure 10:**
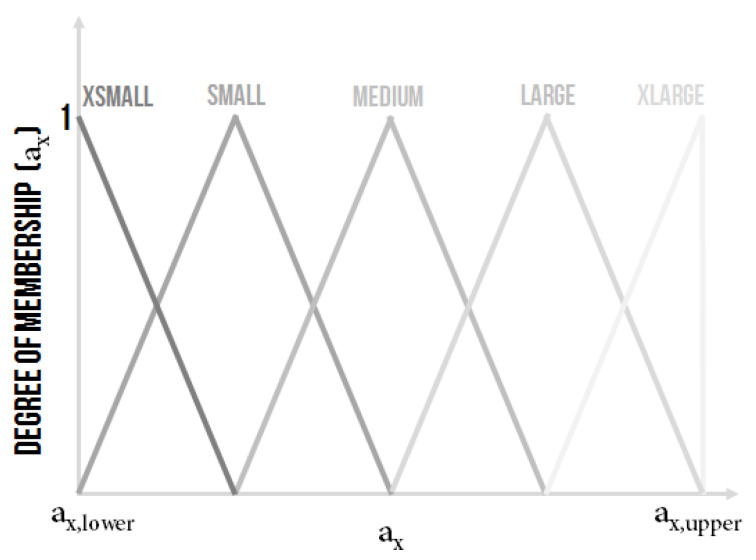
The shape of the membership functions of linguistic values for the input linguistic variable $a_{2}$, $a_{1}$ and $a_{0}$.

**Figure 11:**
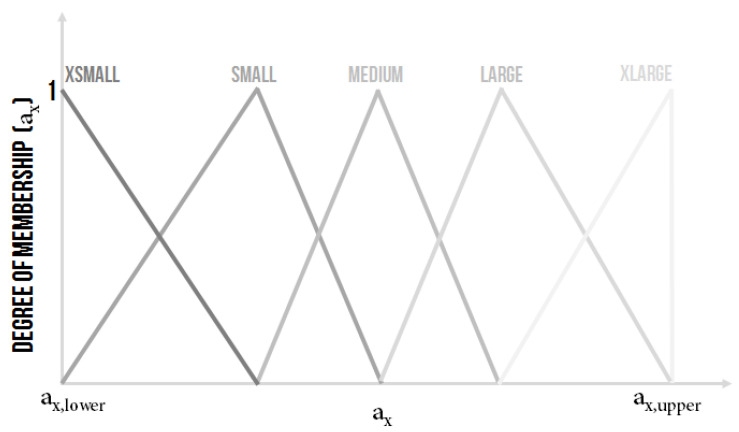
The other possible shape of the membership functions of linguistic values for the input linguistic variable $a_{2}$, $a_{1}$ and $a_{0}$.

The constants from the denominator of the transfer function of the controlled system $a_{2}$, $a_{1}$ and $a_{0}$ were the inputs variables of the ES2 which, as it is further explained, were used for identifying the parameters of a classical PID controller. It was decided that:

- $a_{2}\in \langle a_{2,lower},a_{2,upper}\rangle$,
- $a_{1}\in \langle a_{1,lower},a_{1,upper}\rangle$,
- $a_{0}\in \langle a_{0,lower},a_{0,upper}\rangle$,

with the possible graphical representation in fuzzy sets depicted in Figure 10 or in Figure 11, where, for every linguistic variable, it was proposed to have five linguistic values XSmall (XS), Small (S), Medium (M), Large (L) and XLarge (XL).

In the case presented, five linguistic values for every linguistic variable were used, the description of the shape of the linguistic values of the linguistic variable $a_{2}$ was as follows:

- XSmall (XS)–$\left[0\;0\;6\right]$,
- Small (S)–$\left[0\;6\;11\right]$,
- Medium (M)–$\left[6\;11\;16\right]$,
- Large (L)–$\left[11\;16\;22\right]$,
- XLarge (XL)–$\left[16\;22\;22\right]$.

The description of the shape of the linguistic values of the linguistic variable $a_{1}$ was as follows:

- XSmall (XS)–$\left[0\;0\;5\right]$,
- Small (S)–$\left[0\;5\;10\right]$,
- Medium (M)–$\left[5\;10\;15\right]$,
- Large (L)–$\left[10\;15\;20\right]$,
- XLarge (XL)–$\left[15\;20\;20\right]$.

The description of the shape of the linguistic values of the linguistic variable $a_{0}$ was as follows:

- XSmall (XS)–$\left[0\;0\;9\right]$,
- Small (S)–$\left[0\;9\;14\right]$,
- Medium (M)–$\left[9\;14\;19\right]$,
- Large (L)–$\left[14\;19\;28\right]$,
- XLarge (XL)–$\left[19\;28\;28\right]$.

The shape and the range of the linguistic values of the linguistic variable $a_{2}$, $a_{1}$ and $a_{0}$ were subject to an expert decision.

#### 2.3.2. Controlling System Design Output

The next part focuses on determining the parameters of conventional PID controllers using any appropriate design methods for every combination of the linguistic value of $a_{2}$, $a_{1}$ and $a_{0}$. The values where these fuzzy sets have the maximal value of the membership function should be used as the values of $a_{2}$, $a_{1}$ and $a_{0}$. For every combination, the crisp values of the constants of *K*, $T_{i}$, and $T_{d}$ should be calculated.

As it was mentioned before, three versions of ES2 were considered. The first version was based on the Ziegler–Nichols (ZN) step response method, the second one was based on a combination of Ziegler–Nichols methods (the combination of the frequency response method and the step response Ziegler–Nichols design method) [46] and the last one was based on the possible modification of the Ziegler–Nichols step response method—the Chien, Hrones, and Reswick (CHR) setpoint response design method with a 0% overshoot [1] in a variant with a 0% overshoot (for more information see [31]). These three conventional methods were used to determine the parameters of the conventional PID controller (*K*, $T_{i}$, and $T_{d}$) for systems with all combinations of $a_{2}$, $a_{1}$ and $a_{0}$. In total, 125 crisp values (a single element fuzzy set) for each output value of *K*, $T_{i}$, and $T_{d}$ and for each version were created (the entire table was not included, but it could be easily computed).

#### 2.3.3. Controlling System Design Knowledge Base

Using the information mentioned above, the core of the knowledge base was created. The crisp values of *KKNOW*, *TIKNOW* and *TDKNOW* represent parameters of the classical PID controller with a transfer function in a form of:

(8) $$G_{R}\left(s\right)=KKNOW\left(1+\frac{1}{TIKNOW\cdot s}+TDKNOW\cdot s\right).$$

The definition of the linguistic IF-THEN rules of the Takagi–Sugeno type [47,48] is as follows:

$$R_{r}:\mathrm{If}\left(x_{a_{2}}\mathrm{is}{a_{2}}_{r}\right)\mathrm{and}\left(x_{a_{1}}\mathrm{is}{a_{1}}_{r}\right)\mathrm{and}\left(x_{a_{0}}\mathrm{is}{a_{0}}_{r}\right)\mathrm{then}$$

$$\mathrm{then}\left(KKNOW_{r}=K_{r}\right)\mathrm{and}\left(TIKNOW_{r}=T_{i_{r}}\right)\mathrm{and}\left(TDKNOW_{r}=T_{d_{r}}\right),$$

where r=1,2,⋯,R is a number of the rule.

A fragment of the rule may appear as follows:

⋮

10. $\mathrm{If}\left(a_{2}\mathrm{is}M\right)\mathrm{and}\left(a_{1}\mathrm{is}S\right)\mathrm{and}\left(a_{0}\mathrm{is}S\right)\mathrm{then}\left(KKNOW_{r}\;=\;K_{10}\right)\mathrm{and}\left(TIKNOW_{r}\;=\;T_{i_{10}}\right)\mathrm{and}\left(TDKNOW_{r}\;=\;T_{d_{10}}\right)$
11. $\mathrm{If}\left(a_{2}\mathrm{is}M\right)\mathrm{and}\left(a_{1}\mathrm{is}S\right)\mathrm{and}\left(a_{0}\mathrm{is}M\right)\mathrm{then}\left(KKNOW_{r}\;=\;K_{11}\right)\mathrm{and}\left(TIKNOW_{r}\;=\;T_{i_{11}}\right)\mathrm{and}\left(TDKNOW_{r}\;=T_{d_{11}}\right)$
12. $\mathrm{If}\left(a_{2}\mathrm{is}M\right)\mathrm{and}\left(a_{1}\mathrm{is}S\right)\mathrm{and}\left(a_{0}\mathrm{is}L\right)\mathrm{then}\left(KKNOW_{r}\;=\;K_{12}\right)\mathrm{and}\left(TIKNOW_{r}\;=\;T_{i_{12}}\right)\mathrm{and}\left(TDKNOW_{r}\;=\;T_{d_{12}}\right)$
13. $\mathrm{If}\left(a_{2}\mathrm{is}M\right)\mathrm{and}\left(a_{1}\mathrm{is}S\right)\mathrm{and}\left(a_{0}\mathrm{is}XL\right)\mathrm{then}\left(KKNOW_{r}\;=\;K_{13}\right)\mathrm{and}\left(TIKNOW_{r}\;=\;T_{i_{13}}\right)\mathrm{and}\left(TDKNOW_{r}\;=\;T_{d_{13}}\right)$

⋮

The inference algorithm of the output value in Takagi–Sugeno models is preformed using the weighted mean value. For the output value of *KKNOW*, it is defined in the following form:

(9) $$KKNOW=\frac{\sum_{r=1}^{R}\left\{\left[\mu_{{a_{2}}_{r}}\left(x_{a_{2}}^{*}\right)*\mu_{{a_{1}}_{r}}\left(x_{a_{1}}^{*}\right)*\mu_{{a_{0}}_{r}}\left(x_{a_{0}}^{*}\right)\right]{KKNOW}_{r}\right\}}{\sum_{r=1}^{R}\left[\mu_{{a_{2}}_{r}}\left(x_{a_{2}}^{*}\right)*\mu_{{a_{1}}_{r}}\left(x_{a_{1}}^{*}\right)*\mu_{{a_{0}}_{r}}\left(x_{a_{0}}^{*}\right)\right]},$$

where $\mu_{{a_{2}}_{r}},\mu_{{a_{1}}_{r}}$ and $\mu_{{a_{0}}_{r}}$ represent the degree of membership of the given input (concrete values of the input variable) $x_{a_{2}}^{*},x_{a_{1}}^{*},x_{a_{0}}^{*}$ to its appropriate fuzzy linguistic value in the antecedent of the $r^{th}$ rule. The outputs of the system were represented by the crisp values (constants) of *KKNOW*, *TIKNOW* and *TDKNOW* and the defuzzification was not required. The algorithm of the inference method was simple and computationally unpretentious.

For the second-orders controlled system with three input linguistic variables, where every linguistic variable has five linguistic values, it is necessary to define 125 rules ($5^{3}$).

## 3. Results

The efficiency of every module of the proposed intelligent controlling system was demonstrated independently because all the modules could be used independently and can also work independently. Then, the simulation of the use of all the modules connected to the entire intelligent controller together was provided. The modules were presented separately, with different parameters, to demonstrate the scope of the use. The parameters of the given simulation were selected randomly, in the possible range of the modules. The evaluation of the proposed methods and numerical experiments are presented (simulated) using the Matlab and Matlab and Simulink software environment, and the identification procedure was also implemented using the C++ programming language. The Matlab and Simulink software environment was chosen for its comprehensiveness, since it is considered a standard, well-known and frequently used instrument for engineering simulation in the area of process controlling and measurements.

### 3.1. Monitoring of Control Process Quality–ES1

**Figure 12:**
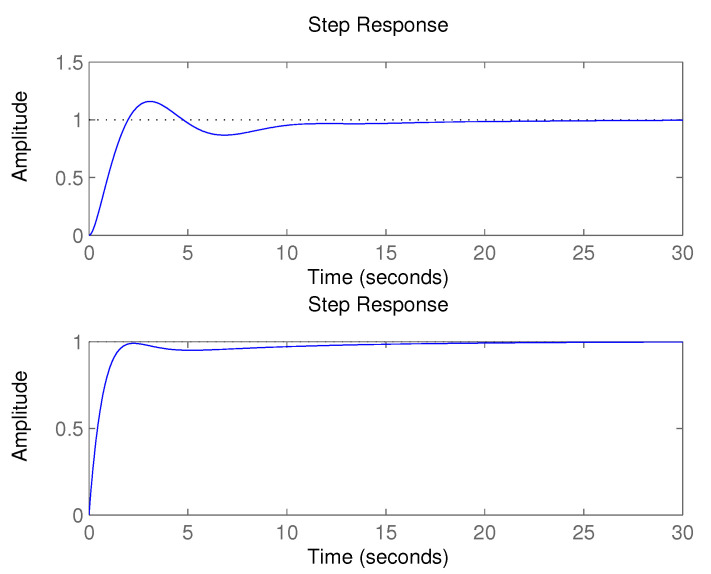
Step response of initial (**upper**) system and changed (**lower**) controlled system according to the simulation of monitoring system [49].

At the beginning, the simulation of the monitoring system was performed with a controlled system of third order using the following transfer function [49]:

(10) $$G_{ES1_{1}}\left(s\right)=\frac{1}{5s^{3}+25s^{2}+8s+3},$$

with an appropriate PID controller. The timing of the step response of the feedback system is shown in Figure 12 (upper). The settling time of the step response was:

(11) $$ST_{ES1_{1}}=18.0444,$$

and relative overshoot:

(12) $$RO_{ES1_{1}}=15.8753.$$

During the controlling process, the controlled system transfer function of the third order was supposed to change to the second order with the following transfer function:

(13) $$G_{ES1_{2}}\left(s\right)=\frac{1}{10s^{2}+7s+2}.$$

The timing of the step response of the feedback loop is shown in Figure 12 (lower). The settling time of the step response now equalled:

(14) $$ST_{ES1_{2}}=12.7590,$$

and the relative overshoot:

(15) $$RO_{ES1_{2}}=0.$$

The score using ES1 was inferred from the inputs:

(16) $$RST_{ES1}=\frac{12.7590}{18.0444}=0.707089,$$

and the relative overshoot:

(17) $$RO_{ES1}=0,$$

and equals:

(18) score=2.66>2.

The value of the score was higher than 2, so the step response had appropriate timing; re-adaptation of the PID controller was unnecessary.

The quality monitoring system was robust enough; the usability was tested for the controlled systems up to the third-order, but there was no reason why it could not work for controlled systems of a higher order. Yet, however, it should be mentioned that stable, controlled systems were taken into consideration here. The next idea stems from the definition of another/other input(s), for which the system was prepared.

### 3.2. Identification of Transfer Function Parameters–IS

The next part deals with the simulation of the identification module (IS), which is going to be demonstrated for the system of the third-order with the following transfer function [49]:

(19) $$G_{IS}\left(s\right)=\frac{s+1}{s^{3}+3s^{2}+3s+4}.$$

Z-transform was used where the sampling period was defined as T=0.25 s; the difference equation of the system was then as follows:

(20) $$\begin{array}{ccc} y_{original}\left(k\right)= & - & \left(-2.31816\right)y\left(k-1\right)-1.83381y\left(k-2\right)-\left(-0.472367\right)y\left(k-3\right)\\ & + & 0.0264792u\left(k-1\right)+0.00182614u\left(k-2\right)-0.0174828u\left(k-3\right). \end{array}$$

After using the identification procedure, the resulting difference equation was determined as:

(21) $$\begin{array}{ccc} y_{identified}\left(k\right)= & - & \left(-2.31758\right)y\left(k-1\right)-1.83278y\left(k-2\right)-\left(-0.471842\right)y\left(k-3\right)\\ & + & 0.0264684u\left(k-1\right)+0.00184947u\left(k-2\right)-0.0174791u\left(k-3\right), \end{array}$$

which was practically the same as (20) with the following best value of the fitness function:

(22) $$J=1.07770\times 10^{-12}.$$

After applying the inverse Z-transform with the same T=0.25 s, the continuous transfer function after identification equalled:

(23) $$G_{identified}\left(s\right)=\frac{-0.0000539209s^{2}+1.00011s+1.00199}{s^{3}+3.00444s^{2}+3.00171s+4.00825}.$$

The approximated system transfer function corresponding to the shape of (20) was determined as:

(24) $$G_{approximated}\left(s\right)=\frac{1.00011s+1.00199}{s^{3}+3.00444s^{2}+3.00171s+4.00825}.$$

**Figure 13:**
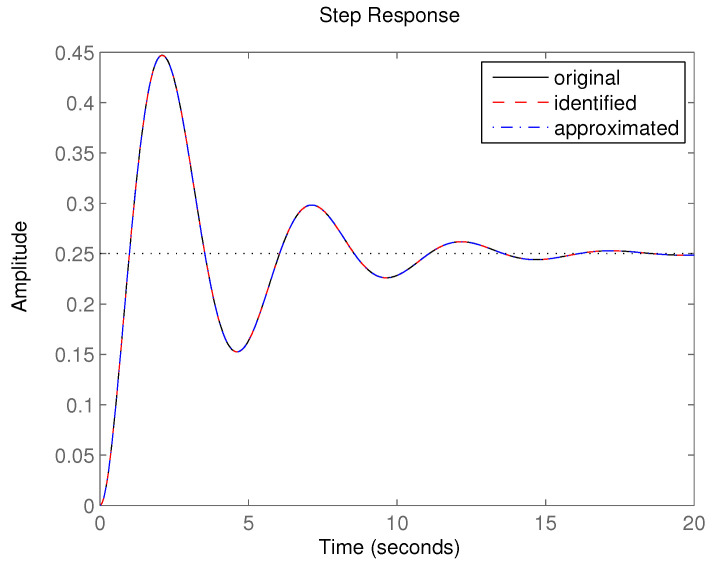
The step response of original, identified and approximated system [49].

To assess the difference in the step response of the original (19), identified (23) and approximated (24) system, see Figure 13. The error calculated as a definite integral of the absolute value of the difference between the original and identified step responses was 0.2946, or 0.03137 between the original and approximated ones. The graphical representation of the step responses of the original, identified and approximated systems was practically the same as the difference was minimal and the curves merged. The computing time for the experiment presented was 6.386 sec based on the data from 97 samples of the input-output values (the simulation time was chosen as 25 s, using sampling period T=0.25 s; three samples were not used as they were incomplete for the system of the third-order because it was necessary to know the data three steps back). The method repeatability was proved by repeating the experiment hundredfold (Table 1).

The proposed method was robust enough and repeatable, as it was proved. The next idea was to shorten the computing time using only the fragments of the signal, which is also especially useful, or to parallelize the identification procedure. Based on our findings, the identification from only the sample of the time response also worked properly, and it was faster [43].

#### Genetic Algorithms Parameters

The use of the genetic algorithms depended on the GA parameters used and the computing power of the equipment employed. All the experiments were performed with the same computing equipment and the same GA setting.

The GA parameters used were set as follows

- Crossover probability—0.9,
- Mutation probability—0.2,
- Population size—31,
- Offspring size—30,
- Selection—rank weighting,
- Crossover—interpolation between two individuals [50],
- Mutation—movement on each dimension by ±0.1× random number.

**Figure 14:**
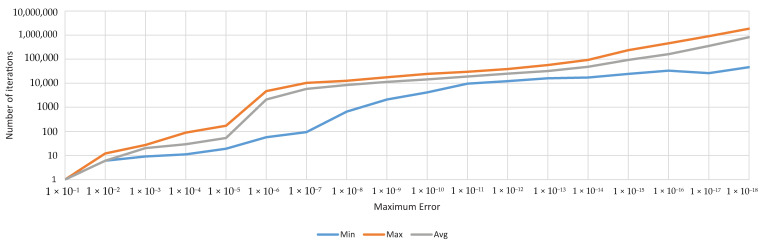
The convergence speed—the accuracy in the point of view of necessary iterations [42].

A combination of the resulting value of fitness function $1\times 10^{-12}$ and 100,000 generations was chosen as the stop criteria for the demonstration of the proposed identification method accuracy. The stop criteria were used independently; if one of them set in, the identification process was stopped. These stop criteria were set to achieve remarkably high accuracy, which could be considered as unnecessary. If it was necessary, according to the accuracy required, the stop criterion could be set in another manner, and the computational time was going to be significantly shorter. For the second-order system, the real computation times were approximately around 1 s for 60,000,000 iterations of the GA conducted on a regular computer with a 2 GHz processor. The convergence speed is shown in Figure 14.

The only criterion for the use of a system of a high order was the computing time, which could be improved using more powerful hardware. The future work in this area is going to focus on the parallelization of the problem. The next question is whether genetic algorithms could be replaced by another method from the artificial intelligence area, such as a bee colony, ant colony algorithms, or particle swarm optimization. All these propositions were viable. It was decided to use genetic algorithms because they were assessed as the best known in the classic industrial area, where doubts about artificial intelligence approaches persisted.

### 3.3. Controlling System Design–ES2

The last separate module to be simulated was the module for the PID controller parameters design. The simulation of ES2 was performed using a system with the transfer function:

(25) $$G_{ES2}\left(s\right)=\frac{1}{3s^{2}+12s+15}.$$

**Figure 15:**
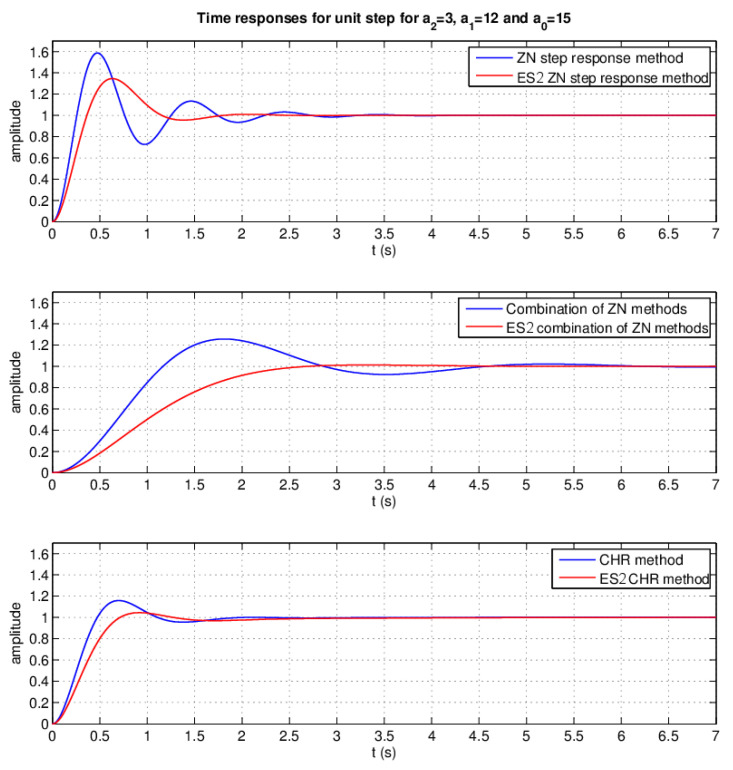
Time responses comparison of the closed feedback loop with a PID controller with parameters determined using the created ES2 and classic appropriate methods for a unit step for $a_{2}$ = 3, $a_{1}$ = 12 and $a_{0}$ = 15 [49].

According to the design of ES2 presented in the paper (see Section 2.3.1), it can be stated that parameter $a_{2}$ was neither XS nor Small, $a_{1}$ was more Small than Medium and $a_{0}$ was Medium rather than Large. ES2 was designed in three versions compared with the traditional PID controller’s corresponding conventional design method (Figure 15)—the Ziegler–Nichols step response method (Figure 15–upper), a combination of Ziegler–Nichols methods (Figure 15–middle) and the Chien, Hrones, and Reswick setpoint response design method with a 0% overshoot in a variant with a 0% overshoot (Figure 15–lower). Comparison of the time response for the unit step of all versions was conducted.

The difference in the overshoot of the time response of a closed feedback control loop for the unit step (Figure 15) was assessed as 24.09%, or 24.375 p.p. and 11.378 p.p. when comparing classic and expert design methods (Table 2) of conventional PID controllers on behalf of expert methods. The difference observed in settling times is shown in Table 2. The settling times in the first two versions were noticeably shorter. In the last option (CHR design method), the settling time was longer, but, with the benefit of the time response, almost without an overshoot.

### 3.4. Intelligent Controller

A general design of the intelligent controller with the artificial intelligence approach is proposed. The intelligent controller concept contained three main parts: quality monitoring, an identification system, and a PID controller design system. The work aimed to show the use and efficiency of artificial intelligence methods in controlling the issue. After verification of the independent modules, all parts (ES1, IS and ES2) were connected, creating an intelligent controlling system according to the schema in Figure 1. The verification focused on demonstrating the efficiency of the proposed intelligent controller.

**Figure 16:**
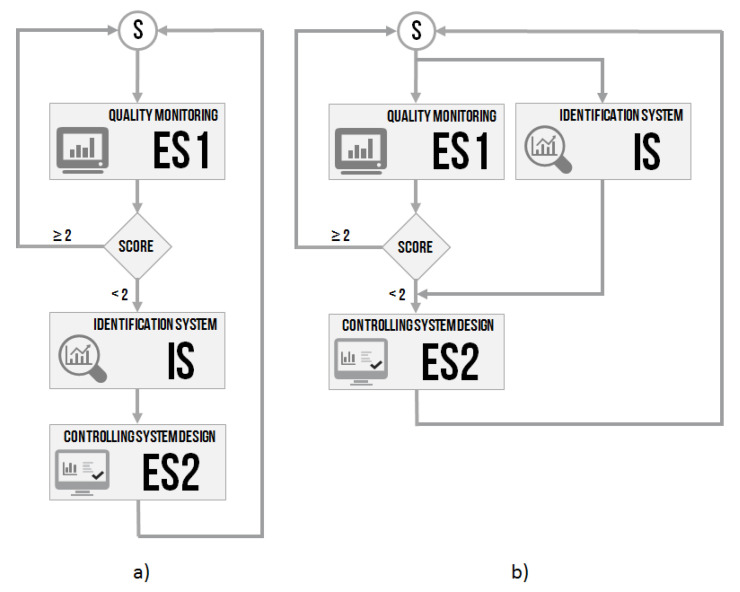
Flowchart of the re-adaptation process in two variants (**a**) with the identification procedure of the parameters of the controlled system after the score evaluation, (**b**) with the continuous system identification.

The re-adaptation strategy using ES1 was briefly described above. For the sake of clarity, the re-adaptation process is shown in Figure 16. The identification procedure (using IS) was continuous (the data were obtained from the sensors continuously, in predefined time samples), so it meant that, in every moment, it should be able to determine the parameters of the controlled system without a significant delay. The ongoing checking of the difference of the required and controlled value (the relative overshoot) of the timing was performed and stored in the memory. In addition, the settling time was measured and stored in the memory after every quick change in the required (step change) or controlled value. After obtaining both values (inputs of ES1), the score was assessed. According to the value of the score,

(26) $$Score\;\;\left\{\begin{array}{ccc} \geq 2 & \;\;\; & \text{do not re-adapt}\\ <2 & \;\;\; & \text{re-adapt} \end{array}\right\},$$

re-adaptation of the PID controller was conducted due to the parameters identified by the IS system. ES2 acted based on the decision made by ES1 on whether the re-adaptation was needed and worked with the knowledge obtained from the identification system about the parameters of the controlled system.

**Figure 17:**
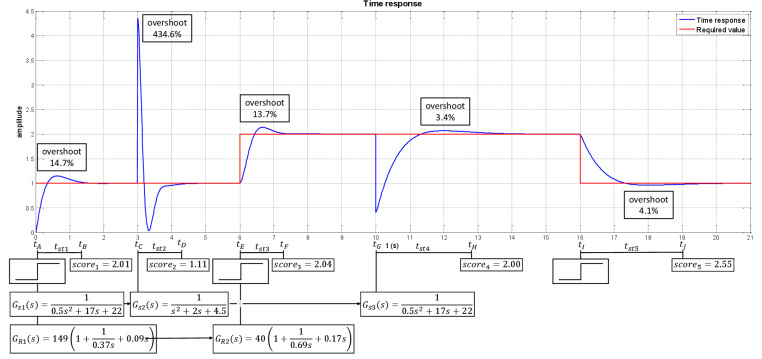
Time course of the simulation of the intelligent controller [49].

The simulation was performed using timing (Figure 17), where all critical moments and information were written. ES2 was used in one of its three versions—the version with the Ziegler–Nichols step response method.

The simulations began with the controlled system (S1) with transfer function

(27) $$G_{S1}\left(s\right)=\frac{1}{0.5s^{2}+17s+22},$$

and its step response in time $t_{A}$. The parameters of the corresponding controller with transfer function

(28) $$G_{R1}\left(s\right)=149\left(1+\frac{1}{0.37s}+0.09s\right),$$

were designed using ES2. In the timing, the overshoot of 14.7% was observed and settling time $t_{st1}$ of 1.4 sec was obtained from measurement. The corresponding score was evaluated using ES1 as

(29) $$score_{1}=2.01>2.$$

The value of $score_{1}$ was bigger than 2, so it means that the timing was assessed as good enough. Then, at time $t_{C}$, the change of the controlled system was performed to

(30) $$G_{S2}\left(s\right)=\frac{1}{s^{2}+2s+4.5},$$

in the timing, the overshoot equalled 434.6%, which can be seen in settling time $t_{st2}=1.2\;\sec$. The quality of process controlling was evaluated as inappropriate using score:

(31) $$score_{2}=1.11<2,$$

as inappropriate. The controlled system was identified using IS with the transfer function:

(32) $$G_{S_{2}ident}\left(s\right)=\frac{1}{s^{2}+2.00122s+4.50034},$$

in 1.232 s (using sampling period T=0.1) with fitness function $J_{S_{2}}=6.13771\times 10^{-10}$. The identification had been stopped before the step change in the required value was performed, but if the identification had not been stopped earlier than the step change was made, the identification would have been stopped by this change in the required value.

The re-adaptation of the parameters of the controller was planned in the next change in the required value, which was introduced in $t_{F}=6$ s. The re-adapted controller had the transfer function in the following form:

(33) $$G_{R2}\left(s\right)=6.1\left(1+\frac{1}{0.58s}+0.14s\right),$$

where the parameters were again designed using ES2 in the version with the Ziegler–Nichols method. The control process was performed with an overshoot equalling 13.7% and settling time $t_{st3}=1.2$ s. The appropriate score calculated by ES1 was:

(34) $$score_{3}=2.04>2,$$

which corresponded to the satisfactory control timing.

Afterwards, at $t_{G}=10\;\sec$, the sudden change in controlled system $G_{S2}$ caused a return to the system which had the same transfer function as $G_{S1}$:

(35) $$G_{S3}\left(s\right)=\frac{1}{0.5s^{2}+17s+22},$$

which was accompanied by the 3.4%-overshoot and settling time $t_{st4}=2.8$ s and

(36) $$score_{4}=2.00,$$

which, according to the proposed procedure (see Figure 16), was assessed as appropriate.

At time $t_{I}$, the step change in the required value was made again; the score using ES1 was inferred as:

(37) $$score_{5}=2.55>2,$$

with the inputs of ES1 settling time $t_{st5}=3$ s and the overshoot = 4.1%. Since $score_{5}$ was bigger than 2, re-adaptation of the PID controller was not necessary, it was adequately compensated by the original controller $G_{R2}\left(s\right)$.

The entire system incorporating all the parts presented worked properly. In the paper, the design is introduced in a general manner so that it can be modified to another family of a controlled system according to the expert’s knowledge.

## 4. Discussion

The paper focuses on the application of a modern soft-computing technology. Conventional mathematical methods and unconventional methods of artificial intelligence are integrated into the design of an intelligent controller system. The solution uses information from sensors performing continuous measurements of indicators of the control process. When dealing with the design of the intelligent controller, several goals were pursued, listed in the introduction of the paper.

Its modularity secures versatility and flexibility of the solution. The sub-subsystem for continuous monitoring of the quality of the control process, the subsystem for ongoing identification of the transient function of the controlled system and the subsystem for continuous adaptation of the PID controller are also usable in other applications.

The process monitoring subsystem module and the controller parameter adaptation subsystem module are fuzzy-logic expert systems; their language models are open for extension by other expert rules. Language models are easily understandable to the users.

Language oriented expert modules use Mamdani and Takagi–Sugeno inference mechanisms. Their algorithms are relatively simple. Similar programs are not computationally intensive and contribute to minimizing the required computing power. For this reason, a simple optimization genetic algorithm is also used in the module of continuous identification of the controlled system. Computing modules, as well as an intelligent global controller, can thus be part of embedded control systems. The simplicity of the solution also supports its use in the school teaching process.

The original solution of Mamdani and Takagi–Sugeno fuzzy modules is the choice of structures of their language models, determination of structures and parameters of fuzzy values of their language variables and expert formulation of language rules by their knowledge base. The use of genetic optimization algorithms in the module of continuous system identification is also original.

When designing the system, the control of higher-order systems was considered. The procedures of individual modules and their integration in the intelligent controller system were programmatically implemented in the Matlab and Simulink system. The correctness of the proposed solutions was simulated by testing both modules and their integration in the intelligent controller system.

The effectiveness of artificial intelligence tools was demonstrated by comparing the results of re-adaptation of PID controller parameters. Three conventional adaptation methods were tested, namely the simple Ziegler–Nichols method, the combined Ziegler–Nichols method and the Chien–Hrones–Reswick method. Their mathematical and fuzzy-language models programmatically implemented all three methods. In all three cases, the application of language models was more successful.

In the further development of the presented intelligent controller system, attention will be paid to its implementation in a microprocessor embedded systems.

## Figures and Tables

**Table 1:** Exploratory statistics corresponding to hundredfold repeating of simulation [49].

		$Y_{1}$	$Y_{2}$	$Y_{3}$	
original		−2.31816	1.83381	−0.472367	
	mean	−2.31318	1.82481	−0.467787	
	median	−2.31390	1.82610	−0.468444	
identified	std	0.00341	0.00618	0.003142	
	min	−2.31769	1.79844	−0.471932	
	max	−2.29859	1.83297	−0.454380	
		$U_{1}$	$U_{2}$	$U_{3}$	**Fitness Function Value**
original		0.0264792	0.00182614	−0.0174828	
	mean	0.0262743	0.00210919	−0.0174222	$1.20482\times 10^{-10}$
	median	0.0263552	0.00203472	−0.0174471	$6.02287\times 10^{-11}$
identified	std	0.0002218	0.00023706	0.0001634	$1.80597\times 10^{-10}$
	min	0.0252438	0.00182211	−0.0180518	$1.07770\times 10^{-12}$
	max	0.0266417	0.00311891	−0.0168724	$1.24241\times 10^{-09}$

**Table 2:** Overshoot and settling time in percent of step response of controlled system with parameters $a_{2}$ = 3, $a_{1}$ = 12 and $a_{0}$ = 15 [49].

Design Method		Overshoot (%)	Settling Time (2% Standard) (s)
ZN step response method	classic	58.69	2.5934
	expert	34.60	1.6355
Combination of ZN methods	classic	25.72	5.4623
	expert	1.345	2.4214
CHR method	classic	15.86	1.6975
	expert	4.482	2.1660

## Abbreviations

The following abbreviations are used in this manuscript:

A	Identificator for value Appropriate
CHR	Chein, Hrones and Reswick
COA	Center of Area
e(t)	Control error
ES	Expert System
ES1	Monitoring Expert System
ES2	Controlling System Design
F	Identificator for value Faster
G	Transfer Function
GA	Genetic Algorithms
H	Identificator for value High
IS	Identification System
J	Fitness Function
K	Gain of Proporcional Component
L	Identificator for value Low and Large
M	Identificator for value Medium
MO	Maximal Overshoot
O	Identificator for value Optimal
PID	Proportional–Integral–Derivative
RO	Relative Overshoot
RST	Relative Settling Time
s	Laplace Operator
S	Identificator for value Slower and Small
sec	Time unit second—for clear usage it is not used s, which is used for Laplace Operator
ST	Settling Time
T	Sampling Period
$T_{d}$	Time Constant of Derivator
TDKNOW	Time Constant of Derivator obtained by Expert System
$T_{i}$	Time Constant of Integrator
TIKNOW	Time Constant of Integrator obtained by Expert System
u(t)	Control variable
U	Laplace Image of Control Variable (System Input)
w(t)	Setpoint—required variable
XL	Identificator for value XLarge
XS	Identificator for value XSmall
y(t)	Controlled variable
Y	Laplace Image of Process Variable (System Output)
ZN	Ziegler–Nichols
